# An insertion in the coding region of the *myostatin *(*MSTN*) gene affects carcass conformation and fatness in the Norwegian Spælsau (*Ovis aries*)

**DOI:** 10.1186/1756-0500-2-98

**Published:** 2009-06-08

**Authors:** Inger A Boman, Dag I Våge

**Affiliations:** 1 Department of Animal and Aquacultural Sciences, Norwegian University of Life Sciences, Ås, Norway; 2 The Norwegian Association of Sheep and Goat Breeders, Ås, Norway; 3 Centre for Integrative Genetics, Norwegian University of Life Sciences, Ås, Norway

## Abstract

**Background:**

A phenotype of increased muscle mass (IMM) and reduced fat, comparable to reported effects of deleterious mutations in the *myostatin *gene (*MSTN*), has been observed in the Norwegian Spælsau breed. However, the genotyping of five AI rams producing descendants with this phenotype, failed to reveal any of the known functional *MSTN *mutations.

**Findings:**

In the present study, the coding region of the *MSTN *gene was sequenced in a Spælsau ram lamb with this particular phenotype. A one base-pair insertion mutation (*c.120insA*) producing a premature stop codon in amino acid position 49 was identified. The consequence of this mutation is that the bioactive carboxy-terminal end of the protein is not translated, and a completely non-functional myostatin protein is produced. Among the 98 available AI rams of this breed, all five individuals having descendants with this particular phenotype were found to be heterozygous for the *c.120insA *mutation. The probability that these five selected AI rams should be heterozygous carriers of the *c.120insA *mutation purely by chance was calculated to be 3.1 × 10^-7^. In total, 7 AI rams were found to be heterozygous carriers of *c.120insA*. The estimated breeding values (EBVs) for EUROP carcass conformation and fat class for these 7 individuals also points towards a strong phenotypic effect of this mutation.

**Conclusion:**

Based upon the completely deleterious effect this novel *c.120insA *mutation has on myostatin protein function, and the documented phenotypic effect of comparable *MSTN *mutations in Norwegian White Sheep and other species, we conclude that this mutation is the functional explanation underlying the IMM phenotype in Norwegian Spælsau. The allele distribution among the 98 genotyped AI rams support this conclusion, and also suggests that *c.120insA *is the most common reason for this phenotype in the Norwegian Spælsau breed.

## Background

We have recently reported a deletion mutation *c.960delG *in the coding part of the *MSTN *gene in the breed Norwegian White Sheep, which strongly affects carcass conformation and fatness in the homozygous state [1]. In the same study it was revealed that the *MSTN *3'-UTR mutation (*c.2360G>A*) identified in Texel sheep, causing a similar but less profound phenotypic effect [2], segregated in the Norwegian White Sheep population [1]. In the Norwegian Spælsau breed, a similar phenotype of increased muscle mass (IMM) and little fat has been observed. In the present study we have sequenced the coding region of the *MSTN *gene in Norwegian Spælsau, to look for a causal explanation of this trait.

## Methods

### Animals and sample preparation

Muscle samples (approximately 0.25 cm^3^) from a Spælsau ram lamb showing the characteristic increased muscle mass (IMM) phenotype were collected and stored in RNA *later*™ (QIAGEN, Hilden, Germany). The IMM lamb was assessed in a commercial abattoir, with a scoring of 12 for carcass conformation class and 2 for fat class (both on a 15 point scale), according to the EUROP classification in Norway [3]. Average carcass conformation class and fat class for this breed was 6.3 and 5.3, respectively, according to the Norwegian Sheep Recording System (SRS) in 2007. A short description of the Norwegian Spælsau breed can be found at the Web [4].

In addition, semen samples from 98 Spælsau AI rams were collected for DNA isolation. After identifying Spælsau lambs with a carcass conformation class of 13 or better in SRS, five of the 98 AI rams were identified as potential mutation carriers based on high estimated breeding values (EBVs) for carcass conformation and low EBVs for fat. See Eikje et al [5] for details on the Norwegian breeding scheme and calculation of EBVs. Additionally, these five individuals shared a familial relationship to the IMM lambs with three of these being sires, one being a grand-sire and one being a great-great-grand-sire of the IMM lambs. EBVs for all AI rams (updated November 2008) were provided by the Norwegian Association of Sheep and Goat Breeders.

Total RNA was extracted with TRIzol^® ^(Invitrogen, Carlsbad, CA, USA), and the isolated RNA was treated with DNase I (Applied Biosystems). Subsequent synthesis of cDNA followed manufacturer's instructions and combined 1 μg RNA in a 20 μl reaction together with a poly dT-primer and SuperScript™ II Reverse Transcriptase (Invitrogen). Genomic DNA was isolated from semen according to standard protocols.

### Sequencing of the MSTN coding region

The ovine *MSTN *coding region was amplified using two primer pairs (F1083/R0997, F0790/R1566; Table 1) whose design was based on the bovine *MSTN *sequence (NM_001001525.2). DNA was amplified using AmpliTaq Gold^® ^(Applied Biosystems, Foster City, CA, USA) using denaturation for 10 min at 95°C, and 40 cycles of 95°C for 30 sec, 58°C for 30 sec and 72°C for 1.5 min. The resulting F1083/R0997 fragment (864 bp) was cloned into the pGEM^®^-T Easy Vector (Promega, Madison, WI, USA) and sequenced with primers Sp6 and M13, while the F0790/R1566 product (777 bp) was sequenced directly using F0790 and R1566 as sequencing primers. A BigDye^® ^Terminator v3.1 kit and a ABI 3730 instrument (Applied Biosystems) was used for all sequencing.

**Table 1:** Primer sequences.

Name	Direction	Position	Sequence 5'-3'
F1083:	Forward	-128 to -109^a^	5'-TCACTGGTGTGGCAAGTTGT-3'
R0997:	Reverse	716–735^b^	5'-TCCTGGTTCTGGGAAGGTTA-3'
F0790:	Forward	528–547^b^	5'-CATCAAACCCATGAAAGACG-3'
R1566:	Reverse	1285–1304^a^	5'-GGTTAAATGCCAACCATTGC-3'
MAF1:	Forward	81–100^b^	5'-CGAGCAGAAGGAAAATGTGG-3'
MAF2:	Reverse	149–168^b^	5'-TATGGCTTCTAGTCTTGAGG-3'
MAE1:	Reverse	118–140^b^	5'-TTTTGTCTCCACAAGCATGCATT-3'

a, Positions are numbered according to first base of the translation start codon of the bovine *MSTN *gene (NM_001001525.2).

b, Positions are numbered according to first base of the translation start codon of the sheep *MSTN *gene (AM992883).

### Genotyping

All genotyping was performed using the Sequenom MassARRAY platform (SEQUENOM, San Diego, USA). Amplification (MAF1, MAF2) and extension (MAE1) primers are described in Table 1.

## Results

Five AI rams identified as potential carriers of a *MSTN *mutation were tested for the previously reported *c.960delG *[1] and *c.2360G>A *mutations [2], using the assays described by Boman et al [1]. Neither mutation was present in these animals. Messenger RNA was therefore isolated from a Spælsau ram lamb showing the IMM phenotype, and the *MSTN *cDNA sequence from this lamb (GenBank accession no. FM207636) was aligned to the corresponding sequence from an individual known to have normal muscle mass (NMM) (GenBank accession no. AM992883). This alignment revealed a 1 bp insertion at nucleotide position 120; *c.120insA *(numbered according to first base of the translation start codon) in the IMM individual. The insertion of an adenine residue will disrupt the reading frame from amino-acid position 40 and onwards, and generate a premature stop codon at amino-acid position 49. This will generate a significantly truncated protein in the IMM individual compared to the 375 amino-acid protein in the NMM individual.

**Figure 1:**
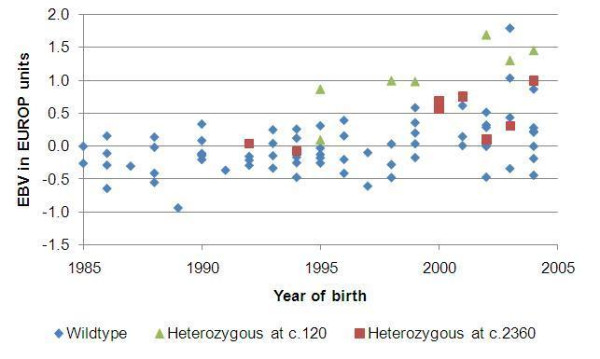
**MSTN-genotypes related to estimated breeding values (EBVs) for carcass conformation class**. *MSTN*-genotypes together with EBVs (15 point scale) for EUROP carcass conformation class for AI Spælsau rams. The EBVs are shown as deviations from the average EBV for carcass conformation class in the reference-population. One ram homozygous for c.2360G>A, with an EVB of 1.1, is not shown in the figure.

**Figure 2:**
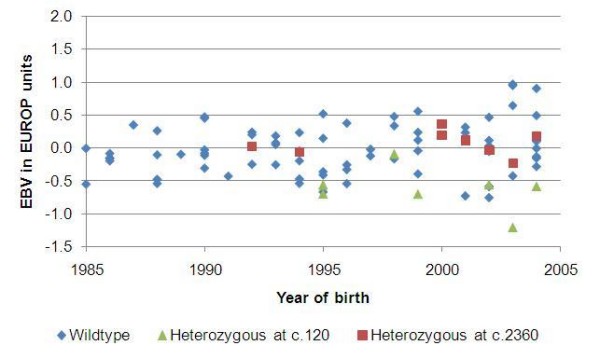
***MSTN*-genotypes related to estimated breeding values (EBVs) for fat class**. *MSTN*-genotypes together with EBVs (15 point scale) for EUROP fat class for AI Spælsau rams. The EBVs are shown as deviations from the average EBV for fat class in the reference-population. One ram homozygous for c.2360G>A, with an EVB of -0.1, is not shown in the figure.

Ninety-eight Spælsau AI rams were genotyped for the *c.960delG *[1], the *c.2360G>A *mutation [2] and the *c.120insA *mutation identified in the present study. Seven individuals were found to be heterozygous for the *c.120insA *mutation, and all five AI rams identified as potential IMM carriers were found in this group. The combinatorial probability of obtaining this outcome purely by chance is 3.1 × 10^-7^. The *c.960delG *mutation was not found in this material, while 8 individuals were heterozygous and 1 homozygous for the *c.2360G>A *mutation. No rams carried both the *c.120insA *mutation and the *c.2360G>A *mutation. In Figures 1 and 2* MSTN*-genotypes, together with EBVs (15 point scale) for EUROP carcass conformation and fat class, respectively, are presented for each AI ram. The EBVs are shown as deviations from the average of the corresponding EBV in the reference population. Fourteen of the older wildtype rams have missing EBVs, and are therefore not included in the figures.

## Discussion

The functional domain of the myostatin protein is composed from amino-acid residues 267–375 [6], therefore homozygous *c.120insA *individuals will not produce a functional myostatin protein. The *c.960delG *mutation in Norwegian White Sheep [1] and comparable frameshift or nonsense mutations in other species like dog [7], and cattle [8], result in a similar phenotype of increased muscle mass and little fat. Although the data linking genotype-phenotype in the present study is limited, the identification of comparable *MSTN *mutations in both sheep and other species have already established the causal link between a non-functional myostatin protein and increased muscle mass and reduced fat. There is no reason to believe that a deleterious mutation like *c.120insA *identified in the present study, should produce a phenotype in Norwegian Spælsau deviating from this picture. The likelihood of *c.120insA *being the causal mutation for the Spælsau IMM phenotype is further strengthen by the fact that the five AI rams with high own EBVs [5] for carcass conformation and fat, and close relationship to IMM lams, were all found to be heterozygous for this mutation in spite of low allele frequency (0.0357) among the AI rams. Also, the genotype/EBV relations shown in Figures 1 and 2 indicate a strong phenotypic effect of *c.120insA*, even in the heterozygous state.

Genotyping revealed that the *c.2360G>A *mutation is also present among the Spælsau AI rams, and this mutation could therefore potentially explain Spælsau IMM phenotypes. However, none of the five rams identified as potential mutation carriers based on phenotypic records have the *c.2360G>A *allele. This might be explained by the fact that while the *c.120insA *is completely deleterious to the myostatin protein, the *c.2360G>A *mutation is reducing the translation rate of the myostatin mRNA [2]. It is therefore reasonable to expect that the most extreme phenotypes will be caused by the deleterious *c.120insA *mutation, rather than the *c.2360G>A *mutation that only causes a reduced concentration of the myostatin protein. This is illustrated in Figures 1 and 2, and agrees with the results obtained by comparing phenotypic effects of the deleterious *c.960delG *mutation and *c.2360G>A *in Norwegian White Sheep [1].

We therefore conclude that *c.120insA *is the functional reason for the pronounced IMM phenotype in the Norwegian Spælsau breed. The 98 genotyped AI rams are considered to be fairly representative of the present gene pool in Norwegian Spælsau. Therefore, we also suggest that *c.120insA *is the most common reason for IMM phenotype in this breed.

## Competing interests

The authors declare that they have no competing interests.

## Authors' contributions

IAB conceived the study and coordinated the sample collection, as well as selected the AI rams based on the SRS. DIV was responsible for the molecular genetics work, analysed the molecular data, and wrote the manuscript together with IAB. Both authors read and approved the final manuscript.
